# The effects of exercise interventions on balance performance in children and adolescents with intellectual disabilities a systematic review and network meta-analysis

**DOI:** 10.3389/fpubh.2026.1796277

**Published:** 2026-08-07

**Authors:** Haibin Gong, Jianan Xu, Wenxian Zhao, Yumin Wang, Meng Yu

**Affiliations:** 1 Graduate School, Shandong Sport University, Jinan, China; 2 College of Physical Education and Sport, Beijing Normal University, Beijing, China; 3 School of Physical Education, Shanxi University, Taiyuan, China; 4 China Institute of Sport Development, Beijing Sport University, Beijing, China; 5 China National Football Academy and China National Basketball Academy, Shandong Sport University, Rizhao, Shandong, China

**Keywords:** adolescent, balance ability, children, exercise, intellectual disabilities

## Abstract

**Objective:**

Intellectual disability is a common developmental disorder in childhood, and deficits in balance ability are particularly prominent in this population. Physical activity is an effective intervention for improving balance. However, the effects of various exercise interventions on balance ability in children and adolescents with intellectual disabilities have not been fully examined.

**Method:**

We conducted a systematic review and network meta-analysis following the PRISMA-NMA guidelines. We searched PubMed, Scopus, VIP, Cochrane Library, CNKI, Wanfang, Web of Science, Embase, and CBM for randomized controlled trials (RCTs) examining exercise interventions for balance in children and adolescents with intellectual disabilities. Risk of bias was assessed using the Cochrane ROB-2 tool.

**Results:**

A total of 3,540 records were identified, of which 22 RCTs met the inclusion criteria. The results showed that the Hopping and Jumping Training Programme (HJP) [MD = 16.60, 95% CI (13.18, 20.02)], Simplified Five-Step Boxing (SFSB) [MD = 2.09, 95% CI (1.43, 3.04)], and Balance Training (BT) [MD = 1.40, 95% CI (0.50, 2.30)] had larger effect sizes compared with control. However, the overall certainty of evidence was low to very low, warranting cautious interpretation.

**Conclusion:**

Exercise interventions may have a beneficial effect on balance ability in children and adolescents with intellectual disabilities. However, due to the low to very low certainty of the evidence, these findings are hypothesis-generating rather than definitive, and strong clinical recommendations cannot be made.

**Systematic review registration:**

https://www.crd.york.ac.uk/PROSPERO/, identifier: CRD420251208290.

## 1 Introduction

Intellectual disability is a common neurodevelopmental disorder in childhood, characterized by limitations in intellectual functioning and adaptive behavior (1). Compared with typically developing peers, children and adolescents with intellectual disabilities are more susceptible to various health problems (2). In China, children and adolescents with intellectual disabilities account for 70% of all children with disabilities, and this population is estimated to be 3.38 million (3, 4). Research indicates that this population exhibits significant delays in motor development, with particularly prominent deficits in balance ability (5). This increases fall risk and causes difficulties in basic daily activities.

Balance refers to an individual's ability to maintain postural stability during static, dynamic, or transitional states. To maintain a balanced posture or achieve a smooth transition between different postures, an individual relies on the integration of visual, vestibular, and proprioceptive sensory inputs to regulate the motor system and produce accurate adaptive responses (6). Due to their intellectual disabilities, children and adolescents with intellectual disabilities often have difficulty coordinating the systems related to balance regulation, which challenges the development of their balance ability. Therefore, exploring safe and effective interventions to improve balance ability in this population has clinical and public health importance.

Various approaches exist to improve balance in children and adolescents with intellectual disabilities, including psychological interventions, virtual reality interventions, and different forms of exercise (7–9). Among these, exercise is considered a core intervention due to its safety and broad applicability. Studies have shown that appropriate physical activities may influence movement-related neural mechanisms (10), promote neural network plasticity (11), and facilitate functional integration in motor-associated regions of the cerebral cortex (12), potentially enhancing central nervous system and proprioceptive processing in this population. Previous research on the effects of exercise in children and adolescents with intellectual disabilities has faced limitations, including single intervention modalities, small sample sizes, and insufficient outcome measures; consequently, the differential effectiveness of adapted exercise interventions remains unclear.

Therefore, to comprehensively evaluate the effects of exercise on balance ability in children and adolescents with intellectual disabilities, this study conducted a systematic review and network meta-analysis of randomized controlled trials (RCTs) that used various exercise interventions targeting balance in this population. Network meta-analysis was chosen over pairwise meta-analysis because it allows simultaneous comparison of multiple interventions and helps identify which exercise modality may be most suitable for specific balance outcomes. This study focuses on exploring the effects of different exercise interventions on balance to provide evidence that informs the selection of suitable exercise interventions for children and adolescents with intellectual disabilities in clinical and rehabilitation practice. It may also help inform personalized exercise programs for this population.

## 2 Method

### 2.1 Protocol and registration

This study was registered with PROSPERO (International Prospective Register of Systematic Reviews; ID = CRD420251208290). This study was conducted in accordance with the Preferred Reporting Items for Systematic Reviews and Meta-Analyses (PRISMA) extension for Network Meta-Analyses (PRISMA-NMA) checklist (Supplementary S13).

### 2.2 Search strategy

The literature search for randomized controlled trials (RCTs) on exercise interventions for balance in children and adolescents with intellectual disabilities was performed on September 20, 2025, across the following databases: PubMed, Scopus, VIP (Chinese Scientific Journal Database), Cochrane Library, CNKI, Wanfang, Web of Science, Embase, and CBM.

We used a combination of Medical Subject Headings (MeSH) terms and free-text terms for the database search, including children, adolescents, intellectual disabilities, balance, randomized controlled trials, and other relevant terms. The detailed search strategy is provided in Appendix S1.

### 2.3 Eligibility criteria

We adopted the “PICOS” framework to define the inclusion criteria of this study: patient: participants were children and adolescents aged 18 years or younger diagnosed with intellectual disabilities, with the diagnosis consistent with the criteria of recognized institutions such as the American Association on Intellectual and Developmental Disabilities (AAIDD) and the Diagnostic and Statistical Manual of Mental Disorders, Fifth Edition (DSM-5). Intervention: Intervention measures included individual training programs, combined training programs, or training programs combined with other related effective treatment methods. Abbreviations for each intervention measure, along with explanations for their classification, are provided in Appendices S2 and S10. Comparison: The control group maintained usual daily activities and regular school physical education without any structured exercise intervention. Outcome: primary Outcome: open-eye Single-leg Standing Test (OSST), Closed-eye Single-leg Standing Test (CSST), Berg Balance Scale (BBS), Timed Up and Go Test (TUGT), the Bruininks-Oseretsky Test of Motor Proficiency, Second Edition (BOT-2), and Force Platform (FP). Secondary Outcome: Sit and Reach Test (TSRT), body mass index (BMI), body fat percentage (BFP), Sit-Ups Test (SUT), and 6-Min Walk Test (6MWT) (replaced the malformed character). Study design: randomized controlled trial (RCT).

### 2.4 Exclusion criteria

(1) Studies focusing solely on specific etiologies of intellectual disabilities [e.g., Down syndrome, autism spectrum disorder (ASD), Williams syndrome, cerebral palsy (CP)]. (2) Participants with any other diseases (e.g., depression, epilepsy, spinal cord injury) that may independently affect balance, or acute/chronic physical conditions [e.g., congenital heart disease, type 2 diabetes mellitus (T2DM), anemia] that interfere with exercise participation. (3) Systematic reviews, conference proceedings, animal studies, books, and observational studies.

Two researchers independently extracted data from the eligible articles, including basic study information (authors, year of publication, title), general participant characteristics of the intervention and control groups (age, sample size), details of exercise training (exercise type, training duration), control measures, and outcome measures. All outcome data were extracted as mean ± standard deviation (SD). Discrepancies encountered during literature screening and data extraction were resolved through discussion between the two researchers; if disagreements persisted, a third professor was consulted.

### 2.5 Data analysis

RevMan 5.4 [The Cochrane Collaboration (Nordic Cochrane Centre), Copenhagen, Denmark] was used to generate risk of bias plots and forest plots for all outcomes. Effect sizes were expressed as mean difference (MD) with 95% confidence intervals (95% CIs); a 95% CI that does not include 0 was considered statistically significant. Heterogeneity among studies was assessed using the *I*^2^ statistic: a fixed-effects model was employed for meta-analysis if *I*^2^ ≤ 50% (indicating low heterogeneity), while a random-effects model was adopted if significant heterogeneity existed (*I*^2^ > 50%).

Network meta-analysis was performed using the “metan” package in Stata 14.0. We first constructed network diagrams to depict the relationships between different exercise interventions, where lines connecting nodes represent direct comparisons between interventions (with line thickness proportional to the number of studies) and node size was proportional to the total sample size. This was followed by the generation of forest plots to present the ranking and effectiveness of each intervention strategy. The surface under the cumulative ranking curve (SUCRA) was used to rank the effectiveness of different exercise types, with larger SUCRA values suggesting higher rankings. Funnel plots were constructed to assess small-study effects and potential publication bias. Given the limited number of studies per comparison (< 10), funnel plot asymmetry tests have low power; therefore, funnel plots are presented for descriptive purposes only without formal statistical testing.

### 2.6 Risk-of-bias assessment

The quality of the included studies was assessed independently by two researchers using the Cochrane Risk of Bias tool for randomized trials (ROB-2). The assessment covered five domains: bias arising from the randomization process, bias due to deviations from intended interventions, bias due to missing outcome data, bias in measurement of the outcome, and bias in selection of the reported result. For each domain, the risk of bias was judged as “low risk”, “some concerns”, or “high risk”. The overall risk of bias for each study was then determined according to the ROB-2 algorithm.

## 3 Results

### 3.1 Search and screening

**Figure 1:**
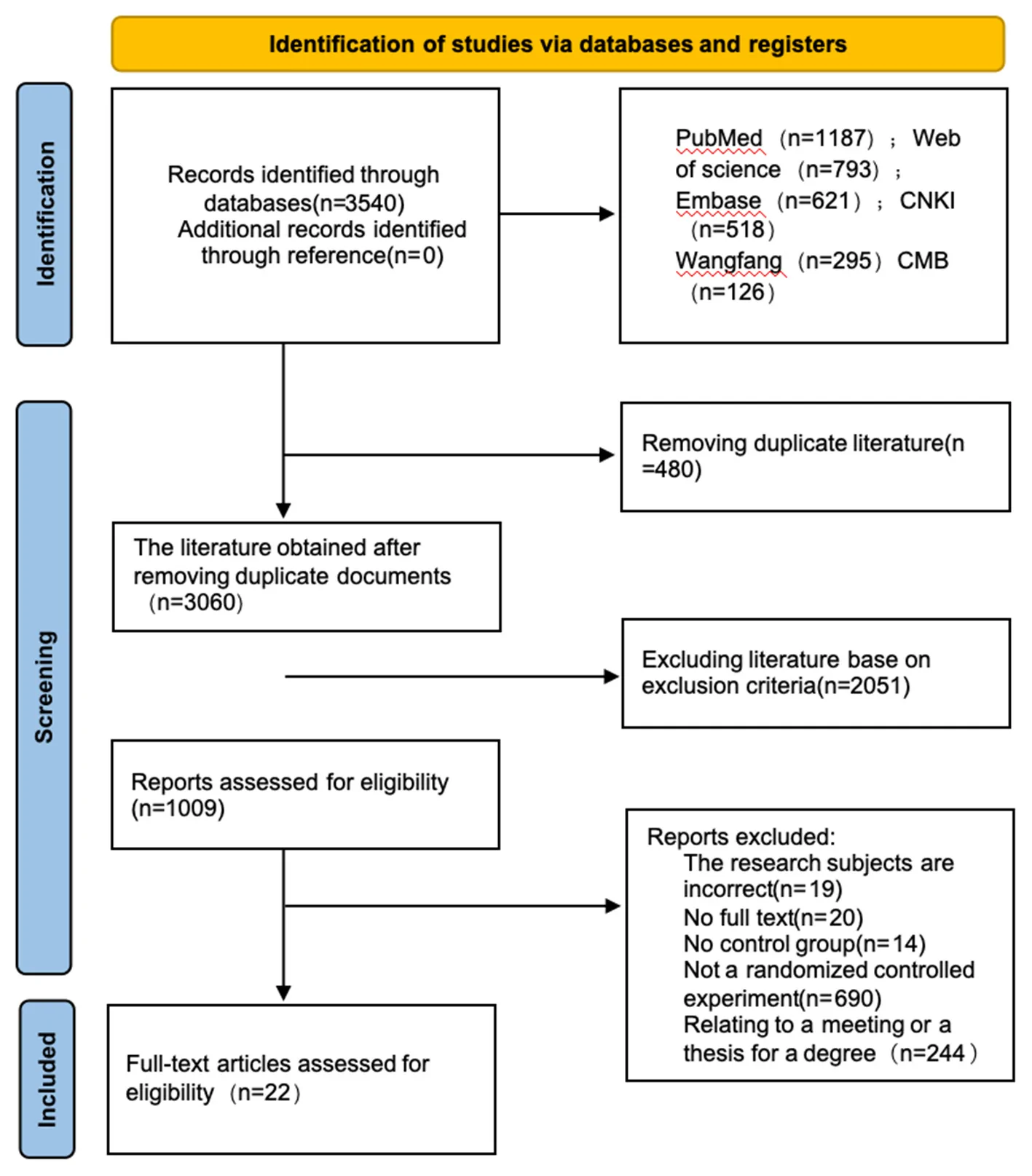
The flow diagram.

We searched both Chinese and English literature databases and obtained a total of 3,540 records. Titles and abstracts were screened manually using NoteExpress. Automated duplicate detection removed 480 records, and the remaining 3,060 records were retrieved for full-text review. Ultimately, 22 studies met the eligibility criteria (13–34). The flow diagram of the study selection process is presented in Figure 1.

### 3.2 Study characteristic

All 22 included studies were designed as randomized controlled trials, involving a total of 879 participants. Table 1 presents the detailed characteristics of the included studies: 502 participants were in the intervention groups and 377 in the control groups. All participants were children or adolescents under 18 years of age. Most studies were two-arm RCTs, with the control groups receiving routine treatment; however, four studies adopted a three-arm RCT design, including arms for routine treatment, aerobic exercise, and floor curling. In all studies, the intervention duration was at least 8 weeks; exercise frequency ranged from 1 to 7 sessions per week, and each session lasted 20 to 60 min. The most common intervention durations were 8 weeks and 12 weeks, and the most common exercise frequency was 2 to 3 sessions per week.

**Table 1:** Table of basic characteristics included in the study.

Trial	Type of disease	Sample size		Age		Intervention		Treatment		Outcome
		Experimental group	control group	Experimental group	control group	Experimental group	control group	Period (w)	Frequency	
Wang et al. (13)	moderate mental retardation	16	13	15.25 ± 0.36	15.00 ± 0.39	Aerobic combined with resistance exercise	Regular exercise	11	40min,4/w	OSST CSST BFP
LiYa (14)	Moderate to Mild Intellectual Disabilities	23 16/7(M/W)	21 12/9(M/W)	13.65 ± 2.06	13.71 ± 2.10	Football	Regular exercise	8	80min,2/w	CSST
Dong Dong (15)	Moderate Intellectual Disabilities	11 9/2(M/W)	12 8/4(M/W)	12.09 ± 0.7	11.92 ± 0.9	Simplified Five-step Boxing	Regular exercise	12	35min,3/w	OSST CSST BBS
Bo and Jun (16)	Severe Intellectual Disabilities	13 7/6(M/W)	13 7/6(M/W)	16.46 ± 2.33	17.23 ± 2.10	Aerobic Exercise	Regular exercise	12	35min,5/2w	BMI BFP 6MWT
Veljković, et al. (17)	mild intellectual disabilities	16 11/5(M/W)	16 11/5(M/W)	17.95 ± 1.53	18.12 ± 0.90	Manipulative skills intervention programme	Regular exercise	12	45min,2/w	BOT-2
Yuvaraj (18)	mild intellectual disability	20	20	13.4 ± 1.04	13.65 ± 1.22	Yoga	Regular exercise	8	45-60min,5/w	OSST TSRT BFP
Ozmen et al. (19)	Moderate to Mild Intellectual Disabilities	16	14	10.9 ± 2.0	11.4 ± 2.0	School-Based Cardiovascular-Fitness Training	Regular exercise	10	60min,3/w	BFP
Lau (20)	mild intellectual disability	125 92/33(M/W)	78 54/24(M/W)	12.8 ± 2.8	12.8 ± 2.8	Video game	Regular exercise	12	30min,2/w	BMI BFP BOT-2
Kong (21)	Severe to Mild Intellectual Disabilities	15	RE:11 AE:14	15.0 ± 1.8	RE:14.8 ± 2.1 AE:14.8 ± 1.8	Tai chi	Regular exercise	12	40min,2/w	OSST TSRT BMI SUT 6MWT
Hsu et al. (22)	mild intellectual disability	27 23/4(M/W)	27 23/4(M/W)	16.59 ± 0.56	16.65 ± 0.63	Floor Hockey Intervention	Regular exercise	12	90min,3/w	TSRT BOT-2
Karakaş (23)	mild intellectual disability	10 7/3(M/W)	11 7/3(M/W)	9.19 ± 1.07	9.19 ± 1.07	adapted physical activities	Regular exercise	24	60min,1/w	BOT-2
Kubilay (24)	mild intellectual disabilities	14 5/9(M/W)	14 7/7(M/W)	14.28 ± 5.13	16.71 ± 5.91	Swiss ball	Regular exercise	8	30min,3/w	TSRT TUGT BFP
Haghighi (25)	mild intellectual disability	8	ART:8 art:8	13–17	ART:13-17 art:13-17	aerobic and rebound therapy	Regular exercise	8	30min,3/w	CSST 6MWT
Lin. (26)	moderate intellectual disabilities	14 12/2(M/W)	14 12/2(M/W)	15.63 ± 0.41	15.62 ± 0.48	rope skipping exercise	Regular exercise	8	50min,3/w	TSRT BMI SUT BFP
Zhao et al. (27)	Moderate Intellectual Disabilities	14	RE:14 FC:14	10.79 ± 1.05	RE:10.50 ± 1.02 FC:10.43 ± 1.10	Aquatic Exercise	Regular exercise	12	60min,3/w	BBS
Borji. (28)	mild intellectual disability	10	RE:10 SRP:10	11.4 ± 1.2	RE:12.3 ± 1 SRP:11.5 ± 0.8	hopping and jumping programme	Regular exercise	8	45min,3/w	BBS
Mikolajczyk (29)	Moderate Intellectual Disabilities	14 15/2(M/W)	14 13/4(M/W)	15.06 ± 0.91	15.06 ± 0.91	dual-task functional exercises	Regular exercise	12	45min,3/w	FP
Lee (30)	mild intellectual disability	15 8/7(M/W)	16 9/7(M/W)	16.27 ± 1.17	17.06 ± 1.75	Balance training	Regular exercise	8	40min,2/w	OSST TUGT FP
Azimizadeh (31)	mild intellectual disability	12 0/12(M/W)	12 0/12(M/W)	10.33 ± 0.88	10.83 ± 0.93	Strengthening and Proprioceptve Training Program	Regular exercise	8	60min,3/w	OSST TUGT
Korkusuz and Top (32)	mild intellectual disability	11 6/5(M/W)	11 4/7(M/W)	11.26 ± 2.03	11.33 ± 1.63	attention skills and fine-gross motor skills	Regular exercise	14	40-60min,2/w	BOT-2
Balayi et al. (33)	mild intellectual disability	15 15/0(M/W)	15 15/0(M/W)	10.06 ± 1.83	9.2 ± 1.24	combined physio-hemsball training	Regular exercise	8	60min,3/w	TUGT
Giagazoglou et al. (34)	Moderate Intellectual Disabilities	9 7/2(M/W)	15 7/2(M/W)	10.3 ± 1.6	10.3 ± 1.6	trampoline exercise intervention program	Regular exercise	12	20min,7/w	TSRT FP

### 3.3 Risk of bias assessment and certainty of evidence

**Figure 2:**
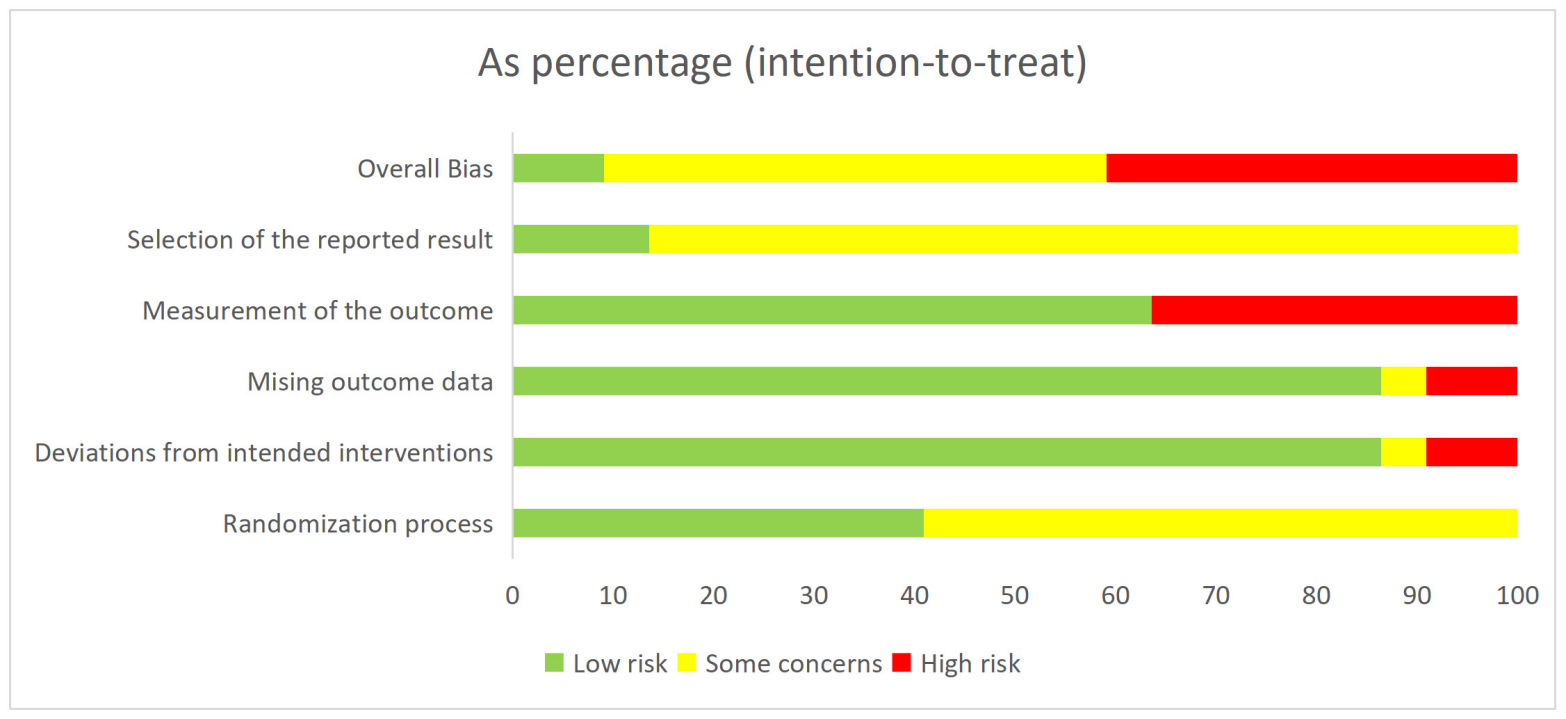
Risk of bias graph.

**Figure 3:**
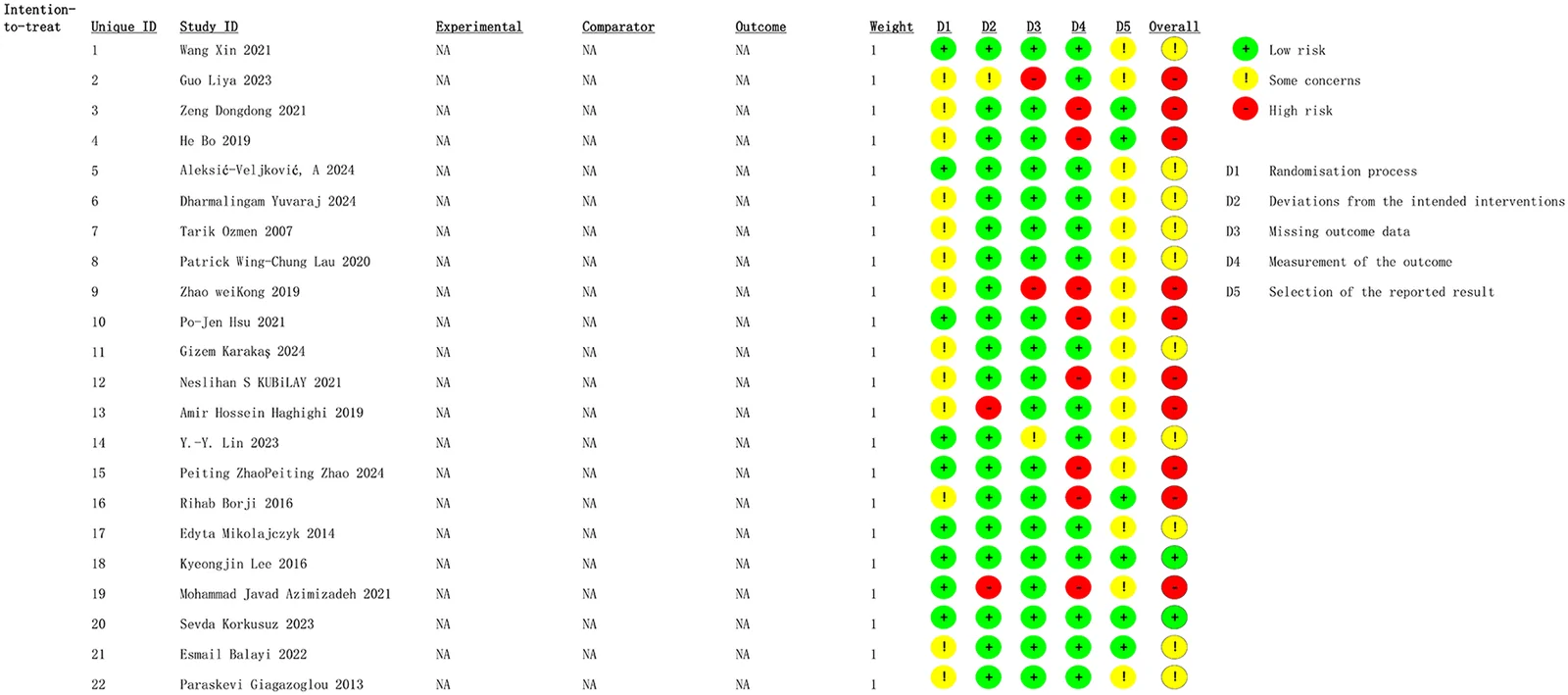
Risk of bias summary.

This study employed the ROB-2 risk of bias assessment tool to evaluate the methodological quality of the included studies. As shown in Figures 2, 3, regarding the randomization process, 9 studies had adequate random sequence generation and allocation concealment with balanced baseline characteristics, judged as low risk; 13 studies were judged as some concerns due to unclear randomization methods or insufficient allocation concealment, with no high-risk studies. Regarding deviations from intended interventions, 19 studies showed good compliance and no obvious protocol deviations (low risk), one study was judged as some concerns due to minor non-compliance or unclear blinding, and 2 studies were judged as high risk due to inconsistent intervention implementation or not using intention-to-treat analysis. Regarding missing outcome data, 19 studies had high completeness (≥95%) with missing data unrelated to true outcomes (low risk); one study was judged as some concerns due to a small amount of unexplained missing data, and 2 studies as high risk due to more extensive or outcome-related missing data. Regarding bias in outcome measurement, 14 studies used unified measurement tools with detailed processes and assessor training, likely reducing bias risk; the other 8 studies had potential subjective bias or incompletely blinded assessors and were judged as high risk. Regarding selection of the reported result, 6 studies were consistent with pre-registered protocols without selective reporting (low risk); 16 studies were judged as some concerns due to incomplete reporting or lack of a pre-specified analysis plan, with no high-risk studies. Therefore, among the 22 included studies, 2 were judged as low risk, 10 as some concerns, and 10 as high risk.

## 4 Meta-analysis

### 4.1 Effectiveness of exercise

**Figure 4:**
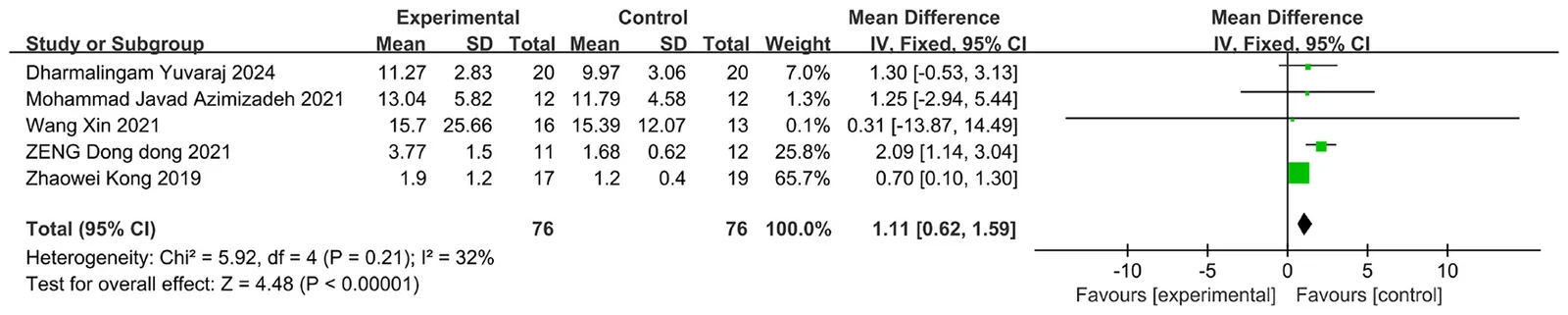
Forest plot of open single-leg stance test.

**Figure 5:**
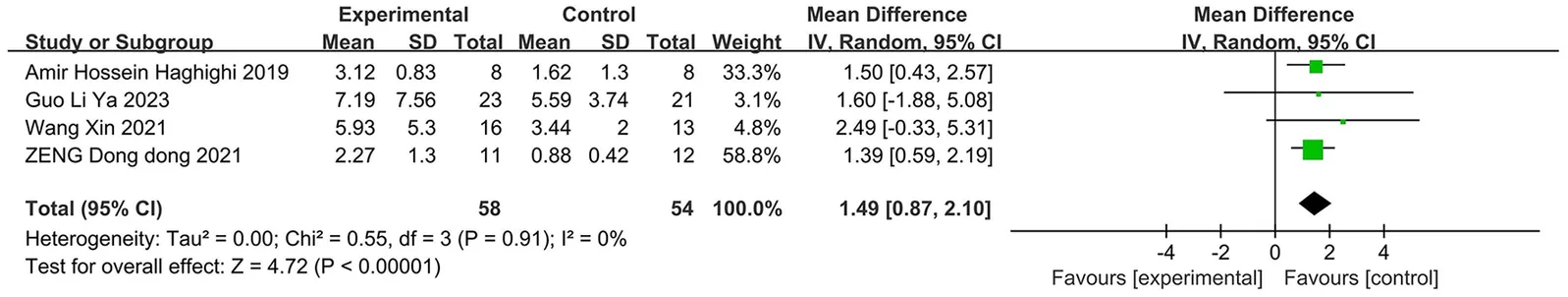
Forest plot of close single-leg stance test.

Single-leg stance is a commonly used indicator for assessing balance ability. Therefore, this study selected OSST and CSST for analysis (Figures 4, 5); other relevant indicators are presented in the Appendix S3. Five studies evaluated the effect of exercise on OSST. The results showed no significant heterogeneity among the studies (Chi^2^ = 5.92, *df* = 4, *P* = 0.21; *I*^2^ = 32%). A fixed-effects model was used for the overall analysis, which revealed a statistically significant difference between the intervention and control groups [MD = 1.11, 95% CI (0.62, 1.59), Z = 4.48, *P* < 0.00001]. Specifically, the mean OSST duration in the intervention group was significantly longer than that in the control group, suggesting that exercise was associated with a positive effect on this outcome measure. Four studies assessed the effect of exercise on CSST. The results showed no heterogeneity (Chi^2^ = 0.55, *df* = 3, *P* = 0.91; *I*^2^ = 0%). A fixed-effects model was employed for the overall effect test, showing a statistically significant difference between the two groups [MD = 1.49, 95% CI (0.87, 2.10), Z = 4.72, *P* < 0.00001]. That is, the mean CSST duration in the intervention group was significantly longer than that in the control group, suggesting that exercise also had a positive effect on this outcome measure.

## 5 Network meta-analysis

### 5.1 Network analysis diagram

**Figure 6:**
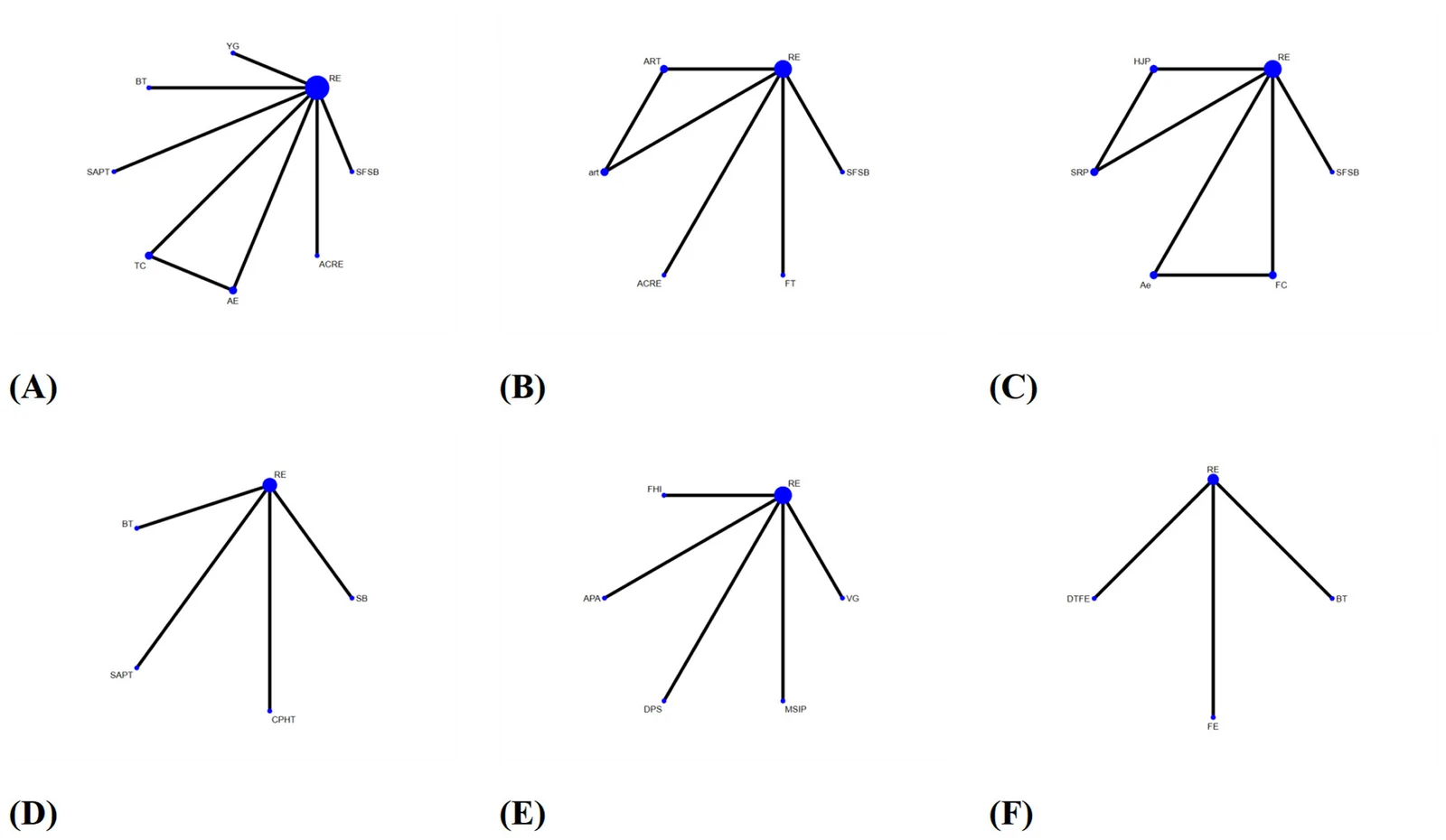
Indicator network diagram. **(A)** OSST. **(B)** CSST. **(C)** BBS. **(D)** TUGT. **(E)** BOT-2. **(F)** FP.

Figure 6 presents the network evidence diagram for the comparisons between different exercise interventions. As illustrated, among the six outcome measures, three formed complete closed loops. Following global and local inconsistency tests for these three measures, all results showed *P* > 0.05, indicating no significant inconsistency between the included studies. Thus, a consistency model was adopted for analysis in this study. The remaining three outcome measures failed to form closed loops, and therefore no relevant inconsistency tests were performed. For the remaining five indicators, the local inconsistency results are presented in Appendix S4.

### 5.2 Primary balance outcomes

#### 5.2.1 OSST

**Figure 7:**
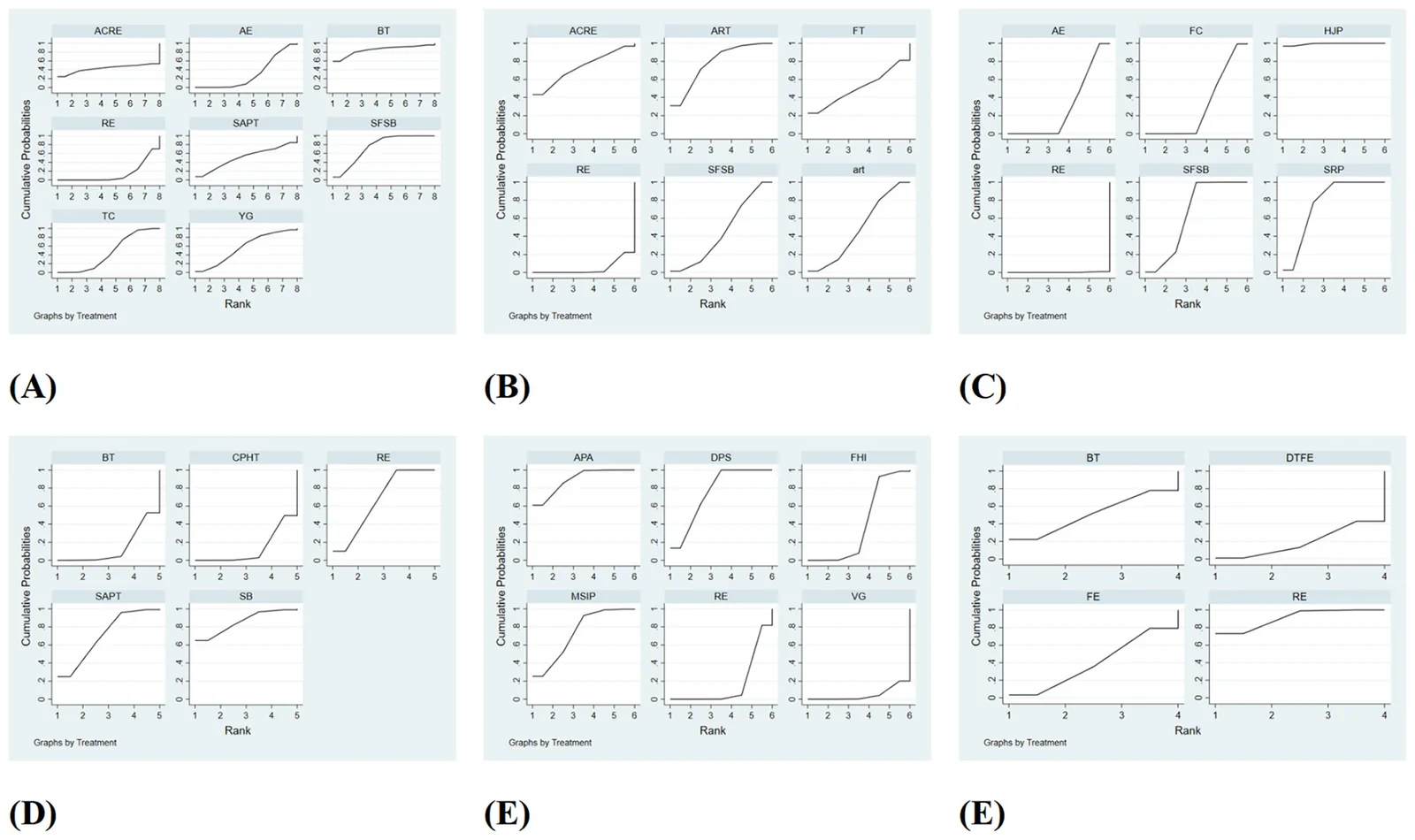
The SUCRA chart of the main indicators. **(A)** OSST. **(B)** CSST. **(C)** BBS. **(D)** TUGT. **(E)** BOT-2. **(F)** FP.

**Figure 8:**
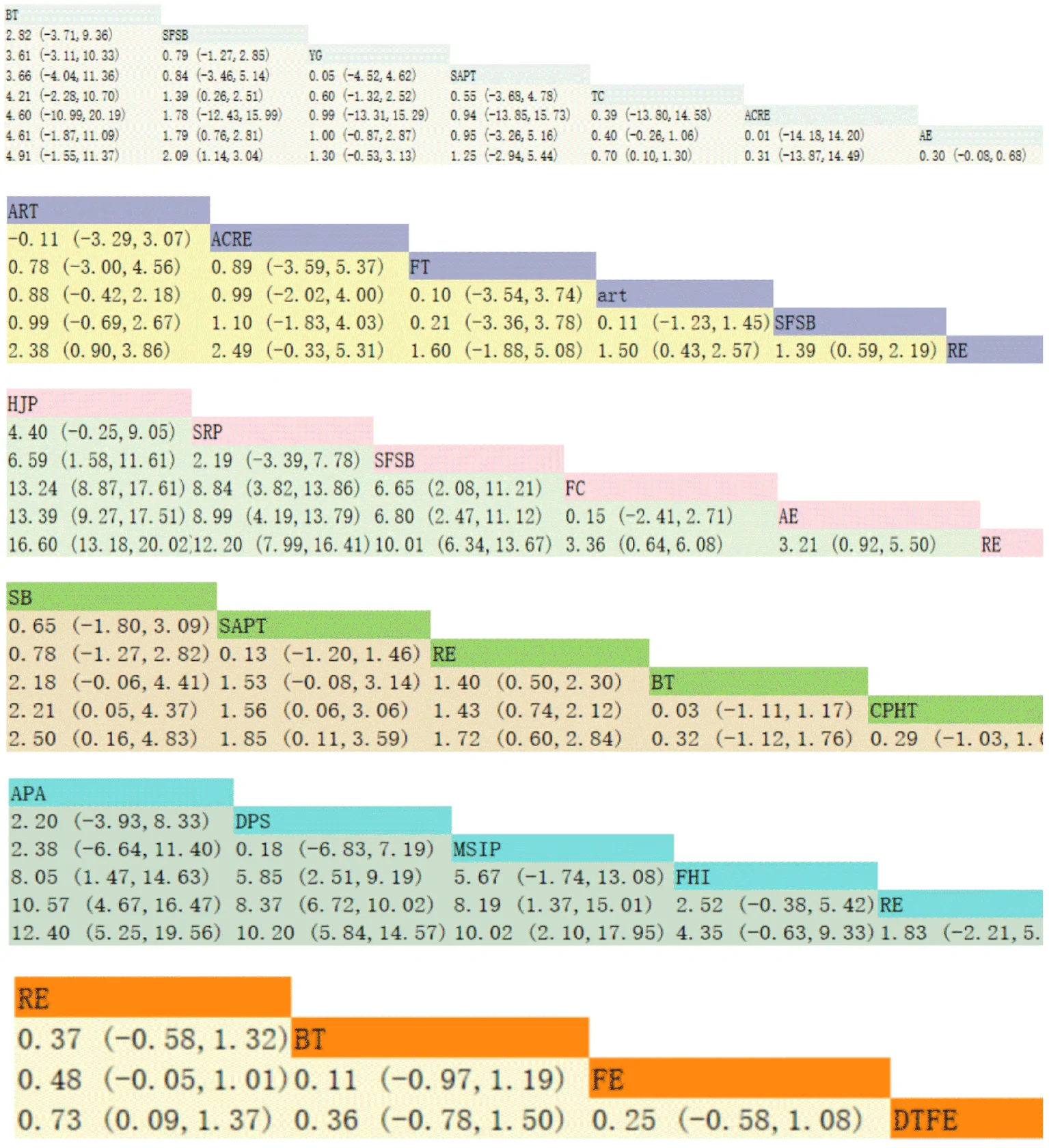
Main indicators' league table.

Five studies were included in the OSST analysis. The baseline forest plot (Appendix S5) showed no statistically significant difference between exercise interventions and control [MD = 0.37, 95% CI (−0.37, 1.12), *P* = 0.33], with moderate heterogeneity (*I*^2^ = 55%). SUCRA (Figure 7) results indicated that SFSB and TC had higher probabilities of being effective. The league table (Figure 8) revealed that compared with RE, both SFSB [MD = 2.09, 95% CI (1.43, 3.04)] and TC [MD = 0.70, 95% CI (0.10, 1.30)] had statistically significant effect sizes, with SFSB showing a larger effect. No established MCID (Minimal Clinically Important Difference) is available for the single-leg standing test in children with intellectual disabilities. Therefore, clinical interpretation of the effect magnitude should be based on the observed mean differences and their confidence intervals, rather than absolute thresholds. Funnel plot analysis suggested no publication bias (Appendix S6). According to the GRADE framework, the overall certainty of evidence was rated as low, downgraded due to risk of bias (limitations in randomization, allocation concealment, and blinding across included studies; Appendix S7). Sensitivity analysis (Appendix S8), which sequentially omitted each study and recalculated the pooled effect using a random-effects model, showed that the combined MD ranged from 0.49 to 2.05—all within the 95% CI of the overall estimate. No individual study substantially altered the direction or magnitude of the effect, indicating that the pooled result for OSST [MD = 1.11, 95% CI (0.62, 1.59)] is robust and not driven by any single trial.

#### 5.2.2 CSST

Baseline comparison (Appendix S5) showed no significant difference between exercise and control [MD = 0.06, 95% CI (−0.24, 0.38), *P* = 0.71; *I*^2^ = 0%). SUCRA (Figure 7) results indicated that ART, ACRE, and FT had the highest probabilities of being effective. Based on four studies, the league table showed that compared with RE, ART [MD = 2.38, 95% CI (0.90, 3.86)], art [MD = 1.50, 95% CI (0.43, 2.57)], and SFSB [MD = 1.39, 95% CI (0.59, 2.19)] had statistically significant effects. Similar to OSST, no MCID has been defined for closed-eye single-leg standing in this population. The MD of 1.49 s [95% CI (0.87, 2.10)] corresponds to a moderate effect size, which may indicate clinically relevant improvement in static balance under sensory-deprived conditions. Funnel plot analysis suggested no publication bias (Appendix S6). The GRADE certainty was low, downgraded for risk of bias (Appendix S7). Sensitivity analysis (Appendix S8) confirmed robustness: recalculated MDs (0.99–2.34) all lay within the 95% CI of the overall estimate [MD = 1.49, 95% CI (0.87, 2.10)], and no single study disproportionately influenced the combined effect.

#### 5.2.3 BBS

Baseline comparison (Appendix S5) showed no significant difference between exercise and control [MD = −0.37, 95% CI (−1.87, 1.13), *P* = 0.63; *I*^2^ = 0%). SUCRA (Figure 7) results indicated that HJP, SRP, and SFSB had the highest probabilities of being effective. Based on three studies, the league table showed that compared with RE, all five interventions—HJP [MD = 16.60, 95% CI (13.18, 20.02)], SRP [MD = 12.20, 95% CI (7.99, 16.41)], SFSB [MD = 10.01, 95% CI (6.34, 13.67)], FC [MD = 3.36, 95% CI (0.64, 6.08)], and AE [MD = 3.21, 95% CI (0.92, 5.50)]—had statistically significant effect sizes, with HJP showing the largest effect. A change of 3.5–6 points on the BBS may be clinically important across multiple patient groups (35). The effect of HJP (MD = 16.60) substantially exceeds this MCID range, suggesting a potentially important clinical improvement. SRP and SFSB also exceeded this MCID range, while FC and AE provided more modest effects. Funnel plot analysis suggested no publication bias (Appendix S6). The GRADE certainty was very low, downgraded for serious risk of bias and serious imprecision (small sample size and wide confidence intervals) (Appendix S7). Sensitivity analysis (Appendix S8) sequentially omitted each study and recalculated the pooled effect. The resulting MDs ranged from 1.67 to 18.06, and although one exclusion widened the CI to cross zero (lower bound −3.28, upper bound 9.87), no single study reversed the direction of the effect; the pooled estimate remained non-significant across all iterations, confirming stability.

#### 5.2.4 TUGT

Baseline comparison (Appendix S5) showed no significant difference between exercise and control [MD = 0.07, 95% CI (−0.93, 1.08), *P* = 0.89; *I*^2^ = 55%). Lower values indicate better performance. SUCRA (Figure 7) results indicated that SB and SAPT had the highest probabilities of being effective. Based on four studies, the league table showed that compared with RE, BT [MD = 1.40, 95% CI (0.50, 2.30)], CPHT [MD = 1.43, 95% CI (0.74, 2.12)], and MDT [MD = 1.72, 95% CI (0.60, 2.84)] had statistically significant effects, with BT showing the smallest MD (i.e., best performance). For the TUGT, MCID estimates range from 0.22 to 5.31 s (36). The statistically significant effects observed for BT (MD = 1.40 s) and other interventions fell within this MCID range, suggesting they may be of potential clinical relevance. Funnel plot analysis suggested no publication bias (Appendix S6). The GRADE certainty was very low, downgraded for risk of bias and imprecision (Appendix S7). Sensitivity analysis (Appendix S8) confirmed robustness: recalculated MDs (−1.68 to −0.78) all lay within the 95% CI of the overall estimate [MD = 0.07, 95% CI (−0.93, 1.08)], and no single study reversed the direction of the effect.

#### 5.2.5 BOT-2

Baseline comparison (Appendix S5) showed no significant difference between exercise and control [MD = −0.82, 95% CI (−2.06, 0.43), *P* = 0.20; *I*^2^ = 0%]. SUCRA (Figure 7) results indicated that APA, DPS, and MSIP had the highest probabilities of being effective. Based on five studies, the league table showed that compared with RE, APA [MD = 10.57, 95% CI (4.67, 16.47)], DPS [MD = 8.37, 95% CI (6.72, 10.02)], and MSIP [MD = 8.19, 95% CI (1.37, 15.01)] had statistically significant effects, with APA showing the largest effect. Responsiveness indices for the BOT-2 in children with intellectual disabilities have been previously examined (36), but specific MCID values are not publicly available. However, the observed improvements for APA, DPS, and MSIP showed large effect sizes, which may indicate clinically meaningful gains. Funnel plot analysis suggested no publication bias (Appendix S6). The GRADE certainty was moderate, downgraded once for imprecision (insufficient sample size; Appendix S7). Sensitivity analysis (Appendix S8) sequentially omitted each study and recalculated the pooled MD using a random-effects model. The recalculated MDs (0.90–9.71) all lay within the 95% CI of the overall estimate [MD = −0.82, 95% CI (−2.06, 0.43)], and no single study reversed the direction or magnitude of the effect, confirming stability.

#### 5.2.6 FP

Baseline comparison (Appendix S5) showed no significant difference between exercise and control [MD = −0.09, 95% CI (−0.47, 0.28), *P* = 0.62; *I*^2^ = 0%). Lower values indicate better performance. SUCRA (Figure 7) results showed that BT and FE had comparable probabilities to RE, while DTFE ranked lowest. Based on three studies, the league table revealed that only DTFE [MD = 0.73, 95% CI (0.09, 1.37)] had a statistically significant effect compared with RE, but the effect direction favored RE (i.e., DTFE was associated with worse performance). Funnel plot analysis suggested no publication bias (Appendix S6). The GRADE certainty was moderate, downgraded one level for imprecision (insufficient sample size; Appendix S7). Sensitivity analysis (Appendix S8) confirmed robustness: recalculated MDs (−0.92 to −0.18) all lay within the 95% CI of the overall estimate [MD = −0.09, 95% CI (−0.47, 0.28)], and no single study reversed the direction of the effect.

### 5.3 Secondary fitness outcomes

#### 5.3.1 6-Min walk test

Baseline comparison showed no significant difference between exercise and control [MD = 16.54, 95% CI (−49.50, 82.58), *P* = 0.62], with substantial heterogeneity (*I*^2^ = 67%; random-effects model). SUCRA results indicated that ART and art had the highest probabilities of being effective. Based on three studies, the league table showed that compared with RE, ART [MD = 232.60, 95% CI (150.30, 314.90)], art [MD = 137.60, 95% CI (31.00, 244.20)], and AE [MD = 77.78, 95% CI (24.94, 130.61)] had statistically significant effects, with ART showing the largest point estimate; TC [MD = 18.57, 95% CI (−72.92, 110.06)] was not significantly different from RE. Funnel plot analysis suggested no publication bias. The GRADE certainty was low, downgraded for serious risk of bias and imprecision. Sensitivity analysis confirmed robustness: recalculated MDs (−33.72 to 233.89) all lay within the 95% CI of the overall estimate [MD = 16.54, 95% CI (−49.50, 82.58)], and no single study reversed the direction of the effect (Appendix S11).

#### 5.3.2 BMI

Baseline comparison showed no significant difference between exercise and control [MD = −1.02, 95% CI (−2.13, 0.10), *P* = 0.07; *I*^2^ = 15%; fixed-effects model]. SUCRA results indicated that AE and RSE had the highest probabilities of being effective, while VG ranked lowest. Based on four studies, the league table revealed that compared with RE, video game-based interventions (VG) had a statistically significant effect [MD = 2.95, 95% CI (1.75, 4.15)], indicating higher BMI in the VG group (i.e., a less favorable effect). Other comparisons (AE, RSE, TC) did not reach statistical significance. No MCID has been established for BMI in this population, but the direction of effect favors non-VG interventions. Funnel plot analysis suggested no publication bias. The GRADE certainty was very low, downgraded for serious risk of bias and very serious imprecision. Sensitivity analysis sequentially omitted each study and recalculated the pooled MD using a fixed-effects model. The recalculated MDs ranged from −3.78 to 1.63, all within the 95% CI of the overall pooled effect [MD = −1.18, 95% CI (−2.29, −0.07)]. No single study exerted disproportionate influence, and the overall effect remained statistically significant across all iterations, consistently favoring the intervention groups, confirming stability (Appendix S11).

#### 5.3.3 Body fat percentage

Baseline comparison (Appendix S5) showed no significant difference between exercise and control [MD = −1.78, 95% CI (−5.38, 1.83), *P* = 0.33; *I*^2^ = 77%; random-effects model). SUCRA results indicated that aerobic exercise (AE) and yoga (YG) had the highest probabilities of being effective, while resistance and sport-based exercises (e.g., RSE) ranked lowest. Based on six studies, the league table showed that compared with RE, none of the interventions (SCFT, AE, YG, ACRE, VG, RSE) had statistically significant effects, as all confidence intervals crossed zero. Funnel plot analysis suggested no publication bias. The GRADE certainty was very low, downgraded for serious risk of bias and serious imprecision. Sensitivity analysis confirmed robustness: recalculated MDs (−5.92 to 2.57) all lay within the 95% CI of the overall estimate [MD = −1.78, 95% CI (−5.38, 1.83)], and no single study reversed the direction of the effect (Appendix S11).

#### 5.3.4 Sit and reach

Baseline comparison (Appendix S5) showed no significant difference between exercise and control [MD = 0.31, 95% CI (−0.40, 1.01), *P* = 0.39; *I*^2^ = 0%; fixed-effects model). SUCRA results indicated that HI and RSE had the highest probabilities of being effective. Based on five studies, the league table revealed that compared with RE, HI [MD = 7.56, 95% CI (2.00, 13.12)] and RSE [MD = 10.07, 95% CI (0.56, 19.58)] had statistically significant effects. Other interventions (FHI, TC, AE, WB, YG) did not reach statistical significance. Funnel plot analysis suggested no publication bias. The GRADE certainty was very low, downgraded for serious risk of bias and serious imprecision. Sensitivity analysis confirmed robustness: recalculated MDs (−1.49 to 4.22) all lay within the 95% CI of the overall estimate [MD = 0.31, 95% CI (−0.40, 1.01)], and no single study reversed the direction of the effect (Appendix S11).

#### 5.3.5 Sit-Ups Test

Baseline comparison (Appendix S5) showed no significant difference between exercise and control [MD = −0.23, 95% CI (−0.56, 0.09), *P* = 0.16; *I*^2^ = 30%; fixed-effects model). SUCRA results showed that none of the interventions had a clearly superior probability of being effective, and all confidence intervals crossed zero. Based on three studies, the league table confirmed that compared with RE, none of the interventions—SB [MD = 1.78, 95% CI (−1.74, 5.30)], TC [MD = 0.20, 95% CI (−0.29, 0.69)], AE [MD = 0.20, 95% CI (−0.42, 0.82), or RSE [MD = 3.58, 95% CI (−2.46, 9.62)]—had statistically significant effects. Funnel plot analysis suggested no publication bias. The GRADE certainty was very low, downgraded for serious risk of bias and very serious imprecision. Sensitivity analysis confirmed robustness: recalculated MDs (−4.91 to 5.79) all lay within the 95% CI of the overall estimate [MD = −0.23, 95% CI (−0.56, 0.09)], and no single study reversed the direction of the effect (Appendix S11).

## 6 Discussion

Through a systematic review and network meta-analysis, this study focused on randomized controlled trials investigating the effects of different exercise interventions on balance ability in children and adolescents with intellectual disabilities. The results suggested that exercise interventions were associated with statistically significant improvements in balance ability. Secondary outcome measures such as BMI also showed some improvements, but these were not integrated into the main conclusions on balance performance. These findings may offer guidance for the rehabilitation of balance ability in children and adolescents with intellectual disabilities.

Balance ability refers to an individual's capacity to maintain postural stability during static standing, dynamic movement, or transitions between postures (37–39). Its regulation involves the integration of sensory inputs from the visual, vestibular, and proprioceptive systems, as well as appropriate motor responses (40–42). The present study suggested that different exercise modalities had differential effects on specific balance dimensions. For example, HJP, APA, and SFSB were reported to show potential benefits in improving balance ability in children and adolescents with intellectual disabilities, which is consistent with previous meta-analyses (5, 43, 44). Furthermore, we identified that different exercises appeared to have targeted effects on distinct aspects of balance. However, given the low to very low certainty of the evidence, these observations remain hypothesis-generating.

Static balance: TC and SFSB may enhance static balance by strengthening the stability of core muscle groups, improving their strength and coordination, and reducing postural sway. This aligns with prior studies demonstrating that core training improves static balance in typically developing children (45, 46).

Dynamic balance and functional mobility: BT interventions may be effective in gait training that specifically targets dynamic center of gravity transfer and step length control. Children with intellectual disabilities often experience dynamic balance impairments due to deficits in motor planning ability (47). BT-based interventions may help improve dynamic balance through repetitive gait pattern practice (48), which might be associated with better motor coordination and postural adjustment.

Comprehensive postural control: integrated exercise interventions such as HJP may be beneficial for multi-muscle group coordination and postural adjustment strategies by enhancing the coordinated force generation of core, lower limb, and other muscle groups (49).

For comprehensive balance improvement measured by the BBS, HJP involves multi-directional jumping and landing activities that repeatedly challenge whole-body coordination and rapid postural adjustments, this type of practice may contribute to the use of multiple postural strategies such as ankle, hip, and stepping strategies (50, 51). For dynamic balance and functional mobility measured by the TUGT, BT focuses on gait training including repetitive practice of center-of-gravity transfer and step length control, such task-specific repetition may be associated with improved automaticity of dynamic balance control (52, 53). For static balance measured by the OSST and CSST, SFSB and TC emphasize slow, controlled movements and sustained isometric postures, which may help improve static balance by supporting the ability to maintain a stable reference of verticality and reducing excessive postural sway (21, 30, 54). For motor proficiency measured by the BOT-2, APA combines balance with fine and gross motor skill training, and the concurrent practice of postural control and manipulative tasks may be beneficial for applying balance ability in functional motor contexts (55). These findings suggest a differentiated applicability of interventions based on individual balance deficits: HJP for comprehensive balance improvement, BT for dynamic balance optimization, SFSB/TC for static balance enhancement, and APA for the coordinated development of balance and motor skills. These suggestions are based on low to very low certainty of the evidence and should be interpreted as hypothesis-generating rather than definitive clinical recommendations.

These findings must be interpreted with caution due to methodological limitations in the included studies. As shown by the ROB-2 assessment, unclear allocation concealment (13 studies), lack of assessor blinding (eight studies at high risk), and small sample sizes threaten the internal validity. These biases likely exaggerate the estimated treatment effects, undermine the stability of the SUCRA rankings, and reduce confidence in the comparative estimates from the network meta-analysis. Consequently, the intervention rankings and effect sizes presented here are hypothesis-generating rather than definitive, and the overall certainty of evidence does not support strong clinical recommendations.

In addition, while the SUCRA rankings provide comparative probability estimates, intervention selection in clinical and educational practice should also consider practical factors such as feasibility, participant age, severity of intellectual disability, available resources, and therapist expertise, rather than relying solely on the ranking probabilities.

## 7 Conclusion

Exercise interventions appear to have positive effects on improving balance function in children and adolescents with intellectual disabilities. However, the overall certainty of evidence is low to very low. Given the limitations of the included studies—small sample sizes, unclear allocation concealment, limited blinding, and possible performance bias—these findings should be interpreted with caution. For improving balance function in this population, some intervention modalities with relatively larger effect sizes may be considered, depending on the outcome measures: for enhancing overall balance stability, HJP-related interventions showed promising results; for optimizing dynamic locomotor balance, BT interventions may be selected; and APA interventions appeared suitable for supporting the coordinated development of balance and motor skills. These findings are hypothesis-generating and need confirmation in future high-quality trials. Future high-quality, adequately powered head-to-head trials are needed to directly compare the effectiveness among different exercise modalities.

## 8 Limitation and further research

The exercise interventions included varied substantially across training mechanisms, intensity, cognitive demands, and motor learning principles, each targeting different balance components. Placing such heterogeneous interventions into a single framework compromises the transitivity assumption of network meta-analysis and reduces the overall certainty of evidence (serious indirectness per GRADE). Therefore, the comparative rankings and effect estimates should be interpreted as hypothesis-generating rather than definitive. Future research should focus on more homogeneous intervention categories (e.g., balance-focused training alone, mind-body exercise alone) to provide more precise evidence.

Several other limitations should be acknowledged. First, key effect modifiers (age, severity of intellectual disability, intervention duration, session length, frequency) were imbalanced across studies, contributing to clinical and statistical heterogeneity; thus intervention rankings should be interpreted with caution. Second, small sample sizes limit statistical power. Third, inadequate reporting of randomization, allocation concealment, and blinding may introduce bias. Fourth, direct comparative evidence is lacking for some outcomes, though random-effects models may partly mitigate this. Fifth, most studies lack long-term follow-up, leaving the sustainability of effects unknown. Future high-quality, large-sample, long-term trials with standardized protocols and clear reporting of intensity and disability severity are needed.

In low-resource settings, interventions requiring minimal equipment (SFSB, TC, basic BT) may be more feasible, as they can be delivered by trained teachers or community health workers without specialized facilities. In contrast, HJP or APA require more space and supervision. For therapists, the choice should consider individual balance deficits (e.g., HJP for comprehensive balance, BT for dynamic balance) while acknowledging the low certainty of the evidence. Schools can integrate SFSB or TC into physical education curricula at low cost. Community programs may adopt BT-based activities for small-group or home-based training. These practical suggestions are preliminary and should be evaluated within local resource constraints.

## Data Availability

The original contributions presented in the study are included in the article/Supplementary material, further inquiries can be directed to the corresponding author.
