# Supplementary material for: The effects of exercise interventions on balance performance in children and adolescents with intellectual disabilities a systematic review and network meta-analysis
Source: Front Public Health. 2026 Aug 7;14:1796277. doi: 10.3389/fpubh.2026.1796277 (PMC13493575; doi:10.3389/fpubh.2026.1796277)
Supplement: Supplementary file 1 [file Supplementary_File_1.docx]

目录

[• S1 The detailed search strategy	3](#_Toc26544)

[1、PubMed	3](#_Toc26632)

[2、Scopus	3](#_Toc4241)

[3、VIP Chinese Scientific Journal Database	3](#_Toc10421)

[4、Cochran Library:	4](#_Toc16322)

[5、China National Knowledge Infrastructure(CNKI)	4](#_Toc24100)

[6、WANGFANG	4](#_Toc4755)

[7、Web of Science	4](#_Toc22518)

[8、Embase	5](#_Toc27503)

[9、CBM	5](#_Toc8972)

[• S2 each intervention measure, along with explanations for their classification	8](#_Toc3914)

[• S3 The forest plot of the remaining 9 indicators	9](#_Toc5567)

[1、Berg Balance Scale	9](#_Toc21384)

[2、The Sit and Reach	9](#_Toc20129)

[3、BMI	9](#_Toc2089)

[4、Sit-up	10](#_Toc17032)

[5、Time up and go test	10](#_Toc26680)

[6、Body fat percentage	10](#_Toc7090)

[7、The Bruininks‒Oseretsky Test of Motor Proficiency™-Second Edition	10](#_Toc27344)

[8、Force Platform	11](#_Toc31554)

[9、6 minute walk test	11](#_Toc24888)

[• S4 All the indicators' tests	12](#_Toc30565)

[• S5 baseline forest plot of outcome indicator（the main resolution indicators）	17](#_Toc29407)

[1、OSST	17](#_Toc17376)

[2、CSST	17](#_Toc20035)

[3、BBS	17](#_Toc8153)

[4、TUGT	18](#_Toc24072)

[5、BOT-2	18](#_Toc15126)

[6、FP	18](#_Toc3018)

[• S6 Funnel plot of 6 outcome indicators (the main resolution indicators)	19](#_Toc5641)

[1、OSST	19](#_Toc9833)

[2、CSST	20](#_Toc15200)

[3、BBS	20](#_Toc28648)

[4、TUGT	21](#_Toc26356)

[5、BOT-2	21](#_Toc14104)

[6、FP	22](#_Toc3399)

[• S7 GRADE evaluation of six outcome indicators (the primary outcome indicator)	23](#_Toc17304)

[1、OSST	23](#_Toc4091)

[2、CSST	23](#_Toc20223)

[3、BBS	24](#_Toc3808)

[4、TUGT	24](#_Toc23590)

[5、BOT-2	24](#_Toc650)

[6、FP	25](#_Toc1514)

[• S8 Sensitivity analysis of six outcome indicators (the primary outcome indicator)	26](#_Toc29845)

[1、OSST	26](#_Toc26851)

[2、CSST	27](#_Toc2810)

[3、BBS	27](#_Toc10206)

[4、TUGT	28](#_Toc1831)

[5、BOT-2	28](#_Toc18717)

[6、FP	29](#_Toc7862)

[• S9 Reasons for including outcome indicators	30](#_Toc23213)

[• S10 6 main indicators and other related images	31](#_Toc756)

[1、OSST(Bar chart and Pairwise Comparisons Forest Plot)	31](#_Toc32334)

[3、BBS(Bar chart and Pairwise Comparisons Forest Plot)	34](#_Toc21917)

[4、TUGT(Bar chart and Pairwise Comparisons Forest Plot)	35](#_Toc16839)

[5、BOT-2(Bar chart and Pairwise Comparisons Forest Plot)	36](#_Toc31862)

[6、FP(Bar chart and Pairwise Comparisons Forest Plot)	37](#_Toc3732)

[• S11 Pictures related to 5 secondary outcome indicators	38](#_Toc4776)

[1、Sit and reach(Bar chart、Comparisons Forest Plot、Funnel Plot、Network Plot、SURCA、Forest plot、GRADE assessment、league table、Baseline comparison、Sensitivity analysis)	38](#_Toc5203)

[2、BMI(Bar chart、Comparisons Forest Plot、Funnel Plot、Network Plot、SURCA、Forest plot、GRADE assessment、league table、Baseline comparison、Sensitivity analysis)	44](#_Toc28173)

[3、Sit-ups test(Bar chart、Comparisons Forest Plot、Funnel Plot、Network Plot、SURCA、Forest plot、GRADE assessment、league table、Baseline comparison、Sensitivity analysis)	49](#_Toc27489)

[4、Body fat percentage(Bar chart、Comparisons Forest Plot、Funnel Plot、Network Plot、SURCA、Forest plot、GRADE assessment、league table、Baseline comparison、Sensitivity analysis)	54](#_Toc8141)

[5、6 minute walk test(Bar chart、Comparisons Forest Plot、Funnel Plot、Network Plot、SURCA、Forest plot、GRADE assessment、league table、Baseline comparison、Sensitivity analysis)	59](#_Toc22910)

[• S12 List of abbreviations	63](#_Toc21246)

# S1 The detailed search strategy

**The retrieval times for all the databases mentioned below are from the establishment date up to September 20, 2025.**

**The language restrictions for all the databases mentioned below are Chinese and English.**

## 1、PubMed

((((((((((((((Disabilities, Intellectual[Title/Abstract]) ) OR (Intellectual Disabilities[Title/Abstract])) OR (Disability, Intellectual[Title/Abstract])) OR (Intellectual Development Disorder[Title/Abstract])) OR (Development Disorder, Intellectual[Title/Abstract])) OR (Development Disorders, Intellectual[Title/Abstract])) OR (Disorder, Intellectual Development[Title/Abstract])) OR (Disorders, Intellectual Development[Title/Abstract])) OR (Intellectual Development Disorders[Title/Abstract])) OR (Mental Retardation[Title/Abstract])) OR (Retardation, Mental[Title/Abstract])) AND (child[Title/Abstract])) OR (children[Title/Abstract])) AND (balance[Title/Abstract])

## 2、Scopus

TITLE-ABS-KEY ( Intellectual Disabilities OR Disabilities , Intellectual OR Disability , Intellectual OR Intellectual Development Disorder OR Development Disorder , Intellectual OR Development Disorders , Intellectual OR Disorder , Intellectual Development OR Disorders , Intellectual Development OR Intellectual Development Disorders OR Mental Retardation OR Retardation , Mental ) AND TITLE-ABS-KEY ( child OR children ) AND TITLE-ABS-KEY ( balance )

## 3、VIP Chinese Scientific Journal Database

(SU=智障 OR 智障儿童 OR 智障学生 OR 智力障碍 OR 智力障碍儿童 OR 智力障碍学生 OR 智力障碍青少年) AND (SU=平衡 OR 平衡能力)

## 4、Cochran Library:

#1 Intellectual Disabilities

#2 (Disabilities, Intellectual):ab,ti,kw or (Disability, Intellectual):ab,ti,kw or (Intellectual Development Disorder):ab,ti,kw or (Development Disorder, Intellectual):ab,ti,kw or (Development Disorders, Intellectual):ab,ti,kw or (Disorder, Intellectual Development):ab,ti,kw or (Disorders, Intellectual Development):ab,ti,kw or (Intellectual Development Disorders):ab,ti,kw or (Mental Retardation):ab,ti,kw or (Retardation, Mental):ab,ti,kw

#3 #1 or #2

#4 child

#5 balance

#6 #3 and #4 and #5

## 5、China National Knowledge Infrastructure(CNKI)

SU=’智障’ OR SU=’智障儿童’ OR SU=’智障学生’ OR SU=’智力障碍’ OR SU=’智力障碍儿童’ OR SU=’智力障碍学生’ OR SU=’智力障碍青少年’ AND SU=’平衡’ OR SU=’平衡能力’

## 6、WANGFANG

题名或关键词:(智障 or 智障儿童 or 智障学生 or 智力障碍 or 智力障碍儿童 or 智力障碍学生 or 智力障碍青少年 ) and 题名或关键词:(平衡 or 平衡能力)

## 7、Web of Science

#1 (TS=Disabilities, Intellectual OR Disability, Intellectual OR Intellectual Development Disorder OR Development Disorder, Intellectual OR Development Disorders, Intellectual OR Disorder, Intellectual Development OR Disorders, Intellectual Development OR Intellectual Development Disorders OR Mental Retardation OR Retardation, Mental)

#2 (TS=children OR child)

#3 (TS=balance)

#4 #1 AND #2 AND #3

## 8、Embase

#1 intellectual AND disabilities

#2**'**disabilities, intellectual':ab,ti OR 'disability, intellectual':ab,ti OR 'intellectual development disorder':ab,ti OR 'development disorder, intellectual':ab,ti OR 'development disorders, intellectual':ab,ti OR 'disorder, intellectual development':ab,ti OR 'disorders, intellectual development':ab,ti OR 'intellectual development disorders':ab,ti OR 'mental retardation':ab,ti OR 'retardation, mental':ab,ti

#3 #1 OR #2

#4 child

#5 balance

#6 #3 AND #4 AND #5

## 9、CBM

((标题=*平衡*) OR 平衡能力) AND ((((标题=*智力障碍学生*) OR 智障儿童) OR 智障学生) OR 智力障碍青少年) OR 智力障碍儿童

| Abbreviation | Full name | Classification reasons |
| --- | --- | --- |
| HJP | Hopping and Jumping Training Programme | It focuses on explosive jumps and consecutive jumps with both legs, emphasizing the elastic recoil of the lower limbs and the rapid release of strength. This has distinct neuromuscular adaptation mechanisms from training such as balance training and endurance training. |
| SRP | Sensorimotor Rehabilitation Programme | It focuses on the integration of multiple senses (vision, vestibular, proprioception) and slow posture control, which is different from the explosive jumps of HJP. The training goals and movement patterns are significantly different. |
| SFSB | Simplified Five-step Boxing | Based on the five-step martial arts form, it focuses on maintaining a stable static posture and the slow movement of the center of gravity, which is different from Tai Chi, balance board training, etc. |
| BT | Balance Training | The core objective is to enhance both static and dynamic balance abilities, typically including specialized exercises such as standing on one foot, standing with eyes closed, and walking on a balance beam, without any obvious strength or jumping elements |
| TC | Tai Chi | It is characterized by slow, continuous and alternating movement of the center of gravity. Its movement rhythm and muscle recruitment pattern are different from ordinary balance training or aerobic exercises. |
| AE | Aerobic Exercise | It mainly involves continuous moderate-intensity activities involving all major muscle groups, aiming to enhance cardiovascular and pulmonary endurance, without specifically focusing on balance or strength. It is fundamentally different from resistance or balance training. |
| ACRE | Aerobic Combined with Resistance Exercise | It contains both aerobic and resistance components, and the training content is complex. Different from single aerobic or single resistance training, it has the characteristics of comprehensive physical training |
| ART | 10 min Aerobic + 20 min Rebound Therapy | Specific combinations (mainly aerobic, supplemented by trampoline rebound training) differ from traditional aerobic or simple rebound therapy in terms of training intensity and intervention methods |
| art | 20 min Aerobic + 10 min Rebound Therapy | Compared with ART, the ratios of aerobic and rebound treatments are different (such as with lower training volume or intensity) |
| FT | Football Training | It involves complex skills such as running, changing direction, kicking the ball, dribbling, etc., and also exercises aerobic capacity, coordination and dynamic balance. It is significantly different from basic aerobic or balance training. |
| SB | Swiss Ball Training | Core and lower limb training is carried out using unstable planes, emphasizing the activation of deep stable muscle groups. Its unstable characteristics are different from those of ground balance training |
| SAPT | Strengthening and Proprioceptive Training | Combining resistance training with proprioceptive stimulation (such as closing eyes and on an unstable surface), but different from pure balance training or pure strength training, it has dual purposes |
| CPHT | Combined Physio‑Hemsball Training | It combines specific therapeutic movements with Hemsball (a throw-and-catch sport), and the training mode is unique and has not appeared in other interventions |
| MDT | Multi‑Domain Training | Simultaneously performing cognitive tasks and motor tasks and interfering with balance control, its training logic is completely different from that of a single motor intervention |
| APA | Adapted Physical Activities | A diverse activity package specifically designed for individuals with intellectual disabilities, which is extensive and personalized, is different from single-item structured training. |
| DPS | Dual‑task Functional Exercises | It is clearly stipulated that the subjects should complete the additional cognitive tasks simultaneously while performing the functional movements. |
| MSIP | Manipulative Skills Intervention Programme | It focuses on fine/gross operation skills such as hand-eye coordination, grasping, and throwing and catching, which is different from full-body balance or strength training |
| FHI | Floor Hockey Intervention | Team ball sports, which include dribbling, hitting the ball, and changing directions during running, are fundamentally different from individual training or non-confrontational sports. |
| VG | Video Game active training | Engaging in physical activities through virtual reality or motion-sensing games, characterized by gamification and screen interaction, is different from real physical exercise. |
| FE | Functional Exercises | Imitating daily life actions (shifting, stretching forward, picking up objects), emphasizing balance control in real scenarios, it is significantly different from abstract sports training |
| DTFE | Dual-task Functional Exercises | Similar to DPS, however, due to differences in specific task design, training cycle or intensity, it was treated as an independent intervention arm in the original study and was not combined with DPS. |
| FC | Floor Curling | A low-intensity throwing and sliding sport modified based on the movements of curling, emphasizing upper limb control and lower limb stable support, is different from ball games or gymnastic activities. |
| YG | Yoga | It includes postures, breathing techniques, and meditation, emphasizing the integration of body and mind as well as the maintenance of static/dynamic postures. Its movement patterns and training philosophy are different from those of martial arts or aerobic exercises. |
| RE | Regular Exercise | As a control condition, it is typically the regular physical education classes at school or daily activities, without any specialized structured intervention. Therefore, it serves as a reference point. |

# S2 each intervention measure, along with explanations for their classification

# S3 The forest plot of the remaining 9 indicators

## 1、Berg Balance Scale

## 2、The Sit and Reach

## 3、BMI

## 4、Sit-up

## 5、Time up and go test

## 6、Body fat percentage

## 7、The Bruininks‒Oseretsky Test of Motor Proficiency™-Second Edition

## 8、Force Platform

## 9、6 minute walk test

# S4 All the indicators' tests

| **6 minute walk** |  | **Coef.** | **Std. Err.** | **Z** | **P>\|z\|** | **[95% Conf.Interval]** | |
| --- | --- | --- | --- | --- | --- | --- | --- |
|  | **_y_B** | -232.6032 | 41.99031 | -5.54 | 0.001 | -314.9027 | -150.3037 |
|  |  |  |  |  |  |  |  |
|  | **_y_C** | -113.5727 | 38.93691 | -2.92 | 0.004 | -189.8876 | -37.25772 |
|  |  |  |  |  |  |  |  |
|  | **_y_D** | -251.172 | 62.7856 | -4 | 0.02 | -374.2295 | -128.1145 |
|  |  |  |  |  |  |  |  |
|  | **_y_E** | -154.8268 | 49.89719 | -3.1 | 0.002 | -252.6235 | -57.03009 |
| **Berg Balance Scale** |  | **Coef.** | **Std. Err.** | **Z** | **P>\|z\|** | **[95% Conf.Interval]** | |
|  | **_y_B** | -10.00762 | 1.871008 | -1.35 | 0.066 | -13.67473 | -6.340507 |
|  |  |  |  |  |  |  |  |
|  | **_y_C** | 6.5924 | 2.559173 | 2.58 | 0.085 | 1.576514 | 11.60829 |
|  |  |  |  |  |  |  |  |
|  | **_y_D** | 2.192398 | 2.850327 | 0.77 | 0.442 | -3.39414 | 7.778936 |
|  |  |  |  |  |  |  |  |
|  | **_y_E** | -6.797093 | 2.205527 | -2.08 | 0.061 | -11.11985 | -2.47434 |
|  |  |  |  |  |  |  |  |
|  | **_y_F** | -6.647091 | 2.330115 | -2.85 | 0.091 | -11.21403 | -2.08015 |
|  | | | | | | | |
| **BMI** |  | **Coef.** | **Std. Err.** | **Z** | **P>\|z\|** | **[95% Conf.Interval]** | |
|  | **_y_B** | 2.949814 | 0.6121313 | 4.82 | 0.056 | 1.750059 | 4.14957 |
|  |  |  |  |  |  |  |  |
|  | **_y_C** | 3.239607 | 1.854662 | 1.75 | 0.081 | -0.3954638 | 6.874678 |
|  |  |  |  |  |  |  |  |
|  | **_y_D** | 1.829392 | 1.628338 | 1.12 | 0.261 | -1.362092 | 5.020876 |
|  |  |  |  |  |  |  |  |
|  | **_y_E** | 3.071135 | 1.211166 | 2.54 | 0.011 | 0.6972938 | 5.444977 |
|  | | | | | | | |
| **Body fat percentage** |  | **Coef.** | **Std. Err.** | **Z** | **P>\|z\|** | **[95% Conf.Interval]** | |
|  | **_y_B** | 4.538783 | 1.399906 | 3.24 | 0.001 | 1.795017 | 7.282549 |
|  |  |  |  |  |  |  |  |
|  | **_y_C** | -2.901291 | 4.411833 | -0.66 | 0.511 | -11.54832 | 5.745743 |
|  |  |  |  |  |  |  |  |
|  | **_y_D** | 7.938336 | 2.592414 | 3.06 | 0.002 | 2.857298 | 13.01937 |
|  |  |  |  |  |  |  |  |
|  | **_y_E** | 3.948373 | 4.525392 | 0.87 | 0.383 | -4.921233 | 12.81798 |
|  |  |  |  |  |  |  |  |
|  | **_y_F** | 4.538459 | 4.303805 | 1.05 | 0.292 | -3.896843 | 12.97376 |
|  |  |  |  |  |  |  |  |
|  | **_y_G** | 4.338724 | 1.65198 | 2.63 | 0.009 | 1.100902 | 7.576546 |
|  | | | | | | | |
| **The sit and reach** |  | **Coef.** | **Std. Err.** | **Z** | **P>\|z\|** | **[95% Conf.Interval]** | |
|  | **_y_B** | -2.24805 | 2.155163 | -1.04 | 0.297 | -6.472093 | 1.975992 |
|  |  |  |  |  |  |  |  |
|  | **_y_C** | -1.677885 | 2.579573 | -0.65 | 0.515 | -6.733756 | 3.377986 |
|  |  |  |  |  |  |  |  |
|  | **_y_D** | -3.07794 | 3.401786 | -0.9 | 0.366 | -9.745317 | 3.589438 |
|  |  |  |  |  |  |  |  |
|  | **_y_E** | -9.807492 | 3.564222 | -2.75 | 0.091 | -16.79324 | -2.821745 |
|  |  |  |  |  |  |  |  |
|  | **_y_F** | 7.821841 | 5.308525 | 1.47 | 0.141 | -2.582677 | 18.22636 |
|  |  |  |  |  |  |  |  |
|  | **_y_G** | -2.147879 | 2.186083 | -0.98 | 0.326 | -6.432524 | 2.136765 |
|  |  |  |  |  |  |  |  |
|  | **_y_H** | -2.047879 | 2.179212 | -0.94 | 0.347 | -6.319055 | 2.223297 |
|  | | | | | | | |
| **BOT-2** |  | **Coef.** | **Std. Err.** | **Z** | **P>\|z\|** | **[95% Conf.Interval]** | |
|  | **_y_B** | 1.833312 | 2.063701 | 0.89 | 0.374 | -2.211467 | 5.878092 |
|  |  |  |  |  |  |  |  |
|  | **_y_C** | 4.353249 | 2.540522 | 1.71 | 0.087 | -0.6260825 | 9.33258 |
|  |  |  |  |  |  |  |  |
|  | **_y_D** | 12.40254 | 3.650727 | 3.4 | 0.001 | 5.247245 | 19.55783 |
|  |  |  |  |  |  |  |  |
|  | **_y_E** | 10.20287 | 2.228301 | 4.58 | 0.004 | 5.835476 | 14.57026 |
|  |  |  |  |  |  |  |  |
|  | **_y_F** | 10.02314 | 4043843 | 2.48 | 0.013 | 2.097354 | 17.94893 |
|  | | | | | | | |
| **TUGT** |  | **Coef.** | **Std. Err.** | **Z** | **P>\|z\|** | **[95% Conf.Interval]** | |
|  | **_y_B** | -0.7767947 | 1.044704 | -0.74 | 0.457 | -2.824377 | 1.270787 |
|  |  |  |  |  |  |  |  |
|  | **_y_C** | -2.176743 | 1.141199 | -1.91 | 0.056 | -4.413452 | 0.0599667 |
|  |  |  |  |  |  |  |  |
|  | **_y_D** | -0.6467129 | 1.246356 | -0.52 | 0.604 | -3.089526 | 1.7961 |
|  |  |  |  |  |  |  |  |
|  | **_y_E** | -2.206641 | 1.102742 | -2 | 0.054 | -4.367975 | -453061 |
|  | | | | | | | |
| **closed-eye single-leg standing** |  | **Coef.** | **Std. Err.** | **Z** | **P>\|z\|** | **[95% Conf.Interval]** | |
|  | **_y_B** | -1.389993 | 0.4102696 | -3.39 | 0.069 | -2.194106 | -0.5858789 |
|  |  |  |  |  |  |  |  |
|  | **_y_C** | 0.9900155 | 0.8582078 | 1.15 | 0.249 | -0.6920408 | 2.672072 |
|  |  |  |  |  |  |  |  |
|  | **_y_D** | 0.1100163 | 0.6823637 | 0.16 | 0.872 | -1.227392 | 1.447425 |
|  |  |  |  |  |  |  |  |
|  | **_y_E** | 1.100007 | 1.493864 | 0.74 | 0.462 | -1.827912 | 4.027926 |
|  |  |  |  |  |  |  |  |
|  | **_y_F** | 0.2100178 | 1.8219 | 0.12 | 0.908 | -3.36084 | 3.780876 |
|  | | | | | | | |
| **open-eye single-leg standing** |  | **Coef.** | **Std. Err.** | **Z** | **P>\|z\|** | **[95% Conf.Interval]** | |
|  | **_y_B** | -2.089216 | 0.4863834 | -4.3 | 0.059 | -3.04251 | -1.135922 |
|  |  |  |  |  |  |  |  |
|  | **_y_C** | -0.789138 | 1.051259 | -0.75 | 0.453 | -2.849567 | 1.271291 |
|  |  |  |  |  |  |  |  |
|  | **_y_D** | -0.8391211 | 2.192524 | -0.38 | 0.702 | -5.136388 | 3.458146 |
|  |  |  |  |  |  |  |  |
|  | **_y_E** | -1.389188 | 0.5741923 | -2.42 | 0.628 | -2.514584 | -0.2637913 |
|  |  |  |  |  |  |  |  |
|  | **_y_F** | -1.789186 | 0.5232777 | -3.42 | 0.756 | -2.814791 | -0.7635802 |
|  | **_y_G** | -1.77916 | 7.25224 | -0.25 | 0.806 | -15.99329 | 12.43497 |
| **Force platform** |  | **Coef.** | **Std. Err.** | **Z** | **P>\|z\|** | **[95% Conf.Interval]** | |
|  | **_y_B** | 0.3695566 | 0.4832719 | 0.76 | 0.444 | -0.5776389 | 1.316752 |
|  |  |  |  |  |  |  |  |
|  | **_y_C** | -0.3604397 | 0.581979 | -0.62 | 0.536 | -1.501098 | 0.7802183 |
|  |  |  |  |  |  |  |  |
|  | **_y_D** | -0.1104826 | 0.5532127 | -0.2 | 0.842 | -1.19476 | 0.9737943 |
| **Sit-up Test** |  | **Coef.** | **Std. Err.** | **Z** | **P>\|z\|** | **[95% Conf.Interval]** | |
|  | **_y_B** | 1.780202 | 1.797854 | 0.99 | 0.322 | -1.743528 | 5.303932 |
|  |  |  |  |  |  |  |  |
|  | **_y_C** | 5.56 | 3.555185 | 1.56 | 0.118 | -1.408035 | 12.52805 |
|  |  |  |  |  |  |  |  |
|  | **_y_D** | 1.580046 | 1.814895 | 0.87 | 0.384 | -1.977082 | 5.137175 |
|  |  |  |  |  |  |  |  |
|  | **_y_E** | 1.980046 | 1.825403 | 1.08 | 0.278 | -1.597678 | 5.55777 |

# S5 baseline forest plot of outcome indicator（the main resolution indicators）

## 1、OSST

## 2、CSST

## 3、BBS

## 4、TUGT

## 5、BOT-2

## 6、FP

# S6 Funnel plot of 6 outcome indicators (the main resolution indicators)

## 1、OSST

## 2、CSST

## 3、BBS

## 4、TUGT

## 5、BOT-2

## 6、FP

# S7 GRADE evaluation of six outcome indicators (the primary outcome indicator)

## 1、OSST

## 2、CSST

## 3、BBS

## 4、TUGT

## 5、BOT-2

## 6、FP

# S8 Sensitivity analysis of six outcome indicators (the primary outcome indicator)

## 1、OSST

## 2、CSST

## 3、BBS

## 4、TUGT

## 5、BOT-2

## 6、FP

# S9 Reasons for including outcome indicators

| **outcome indicator** | **Inclusion reasons** |
| --- | --- |
| **The main outcome measure** | |
| OSST | Directly measure the static balance ability under visual feedback |
|  |  |
| CSST | Directly measure the static balance that depends on vestibular/proprioception |
|  |  |
| BBS | Comprehensively assess the static and dynamic balance |
|  |  |
| TUGT | Directly measure dynamic balance and mobility |
|  |  |
| BOT-2 | The balance item in the standardized motor ability test |
|  |  |
| FP | Objectively measure the postural swing |
| Secondary general indicators | |
| BMI  body fat percentage | Body composition affects posture control by changing the center of gravity and proprioceptive feedback |
|  |  |
| Sit-up test  sit and reach | Core muscle endurance and flexibility are supporting factors for balance |
|  |  |
| 6MWT | Measuring functional endurance may be related to dynamic balance during long walks |
|  |  |
| blood pressure | Abnormal blood pressure can affect the stability of standing. |

# S10 6 main indicators and other related images

## 1、OSST(Bar chart and Pairwise Comparisons Forest Plot)

**2、**CSST(Bar chart and Pairwise Comparisons Forest Plot)

## 3、BBS(Bar chart and Pairwise Comparisons Forest Plot)

## 4、TUGT(Bar chart and Pairwise Comparisons Forest Plot)

## 5、BOT-2(Bar chart and Pairwise Comparisons Forest Plot)

## 6、FP(Bar chart and Pairwise Comparisons Forest Plot)

# S11 Pictures related to 5 secondary outcome indicators

## 1、Sit and reach(Bar chart、Comparisons Forest Plot、Funnel Plot、Network Plot、SURCA、Forest plot、GRADE assessment、league table、Baseline comparison、Sensitivity analysis)

| RSE |  |  |  |  |  |  |  |
| --- | --- | --- | --- | --- | --- | --- | --- |
| 7.82 (-2.58,18.23) | YG |  |  |  |  |  |  |
| 9.50 (-0.41,19.41) | 1.68 (-3.38,6.73) | WB |  |  |  |  |  |
| 9.87 (0.34,19.40) | 2.05 (-2.22,6.32) | 0.37 (-2.48,3.22) | AE |  |  |  |  |
| 9.97 (0.43,19.51) | 2.15 (-2.14,6.43) | 0.47 (-2.40,3.34) | 0.10 (-0.48,0.68) | TC |  |  |  |
| 10.07 (0.56,19.58) | 2.25 (-1.98,6.47) | 0.57 (-2.21,3.35) | 0.20 (-0.43,0.83) | 0.10 (-0.62,0.82) | RE |  |  |
| 10.90 (0.08,21.72) | 3.08 (-3.59,9.75) | 1.40 (-4.46,7.26) | 1.03 (-4.17,6.23) | 0.93 (-4.28,6.14) | 0.83 (-4.33,5.99) | FHI |  |
| 17.63 (6.61,28.65) | 9.81 (2.82,16.79) | 8.13 (1.91,14.35) | 7.76 (2.16,13.36) | 7.66 (2.05,13.27) | 7.56 (2.00,13.12) | 6.73 (-0.86,14.32) | HI |

## 2、BMI(Bar chart、Comparisons Forest Plot、Funnel Plot、Network Plot、SURCA、Forest plot、GRADE assessment、league table、Baseline comparison、Sensitivity analysis)

| AE |  |  |  |  |
| --- | --- | --- | --- | --- |
| -0.17 (-4.16,3.83) | RSE |  |  |  |
| 0.12 (-1.93,2.17) | 0.29 (-3.14,3.72) | RE |  |  |
| 1.24 (-1.63,4.11) | 1.41 (-3.12,5.94) | 1.12 (-1.84,4.08) | TC |  |
| 3.07 (0.70,5.44) | 3.24 (-0.40,6.87) | 2.95 (1.75,4.15) | 1.83 (-1.36,5.02) | VG |

## 3、Sit-ups test(Bar chart、Comparisons Forest Plot、Funnel Plot、Network Plot、SURCA、Forest plot、GRADE assessment、league table、Baseline comparison、Sensitivity analysis)

| RSE |  |  |  |  |
| --- | --- | --- | --- | --- |
| 3.58 (-2.46,9.62) | AE |  |  |  |
| 3.78 (-2.23,9.79) | 0.20 (-0.42,0.82) | RE |  |  |
| 3.98 (-2.05,10.01) | 0.40 (-0.07,0.87) | 0.20 (-0.29,0.69) | TC |  |
| 5.56 (-1.41,12.53) | 1.98 (-1.60,5.56) | 1.78 (-1.74,5.30) | 1.58 (-1.98,5.14) | SB |

## 4、Body fat percentage(Bar chart、Comparisons Forest Plot、Funnel Plot、Network Plot、SURCA、Forest plot、GRADE assessment、league table、Baseline comparison、Sensitivity analysis)

| SCFT |  |  |  |  |  |  |
| --- | --- | --- | --- | --- | --- | --- |
| 3.40 (-0.88,7.68) | RE |  |  |  |  |  |
| 3.40 (-5.65,12.45) | 0.00 (-7.98,7.98) | AE |  |  |  |  |
| 3.60 (-1.01,8.21) | 0.20 (-1.52,1.92) | 0.20 (-7.96,8.36) | YG |  |  |  |
| 3.99 (-5.47,13.45) | 0.59 (-7.84,9.03) | 0.59 (-11.02,12.20) | 0.39 (-8.22,9.00) | ACRE |  |  |
| 7.94 (2.86,13.02) | 4.54 (1.80,7.28) | 4.54 (-3.90,12.97) | 4.34 (1.10,7.58) | 3.95 (-4.92,12.82) | VG |  |
| 10.84 (1.59,20.09) | 7.44 (-0.76,15.64) | 7.44 (-4.00,18.88) | 7.24 (-1.14,15.62) | 6.85 (-4.91,18.61) | 2.90 (-5.75,11.55) | RSE |

## 5、6 minute walk test(Bar chart、Comparisons Forest Plot、Funnel Plot、Network Plot、SURCA、Forest plot、GRADE assessment、league table、Baseline comparison、Sensitivity analysis)

| ART |  |  |  |  |
| --- | --- | --- | --- | --- |
| 113.57 (37.26,189.89) | art |  |  |  |
| 154.83 (57.03,252.62) | 41.25 (-34.80,117.31) | AE |  |  |
| 232.60 (150.30,314.90) | 119.03 (64.32,173.74) | 77.78 (24.94,130.61) | RE |  |
| 251.17 (128.11,374.23) | 137.60 (31.00,244.20) | 96.35 (-1.61,194.30) | 18.57 (-72.92,110.06) | TC |

# S12 List of abbreviations

|  | List of abbreviations |
| --- | --- |
| YG | Yoga |
| BT | Balance Training |
| SAPT | Strengthening and Proprioceptve Training Program |
| TC | Tai Chi |
| AE | Aerobic Exercise |
| ACRE | Aerobic combined with Resistance Exercise |
| SFSB | Simplified Five-step Boxing |
| ART | 10min Aerobic and 20min Rebound Therapy |
| art | 20min Aerobic and 10min Rebound Therapy |
| ACRE | Aerobic Combined with Resistance Exercise |
| FT | Football |
| HJP | Hopping and Jumping Training Programme |
| SRP | Sensorimotor Rehabilitation Programme |
| Ae | Aaquatic Exercise |
| FC | Floor Curling |
| SB | Swiss Ball |
| CPHT | Combined Physio-hemsball Training |
| VG | Video Game |
| APA | Adapted Physical Activities |
| DPS | Developing Attention Skills and Physical Activities Enhancing Fine-gross Motor Skills |
| MSIP | Manipulative Skills Intervention Programme |
| DFTE | Dual-task Functional Exercises, |
| TE | trampoline exercise |
